# Meta-Research Perspectives on Verbal Lie Detection

**DOI:** 10.3390/brainsci13030392

**Published:** 2023-02-24

**Authors:** Galit Nahari

**Affiliations:** Department of Criminology, Bar-Ilan University, Ramat Gan 5290002, Israel; galit.nahari@biu.ac.il

**Keywords:** deception detection, verbal lie detection, richness-in-detail, reality monitoring, verifiability approach, meta-research, coding scheme

## Abstract

Verbal lie detection (VLD) research, conducted mainly in the cognitive sciences, examines content-based indicators that differ between truth-tellers and lie-tellers. Following the meta-research approach (examination of the research itself), the current paper aimed to ascertain the current status of VLD research across three components: the search for VLD indicators, VLD coding, and VLD research tracks. It highlights several issues that challenge VLD research; these are discussed, along with suggestions for how to address them. This scrutiny may contribute to a further advancement of the field of VLD research and, as a result, an improvement of VLD practices.

## 1. Introduction

Given the rapidly growing body of research over recent decades in deception detection via the verbal channel, and the significant progress made in both theoretical understanding and applicable developments, it is an opportune time to critically examine verbal lie detection (VLD) research and its implications for practice. Three dimensions of VLD research will be discussed: the search for VLD indicators, VLD coding, and VLD research tracks. Although recent work has dealt with urgent issues for VLD research [1], the focus of the current discussion is on how to promote VLD research in general and not the specific lines of research that should be developed. As such, a meta-research approach was utilized, namely, the study of the research itself: its methods, its reporting, and its evaluation [2].

## 2. The Search for VLD Indicators

Based on the presupposition that lie-tellers differ from truth-tellers in their verbal expression, a primary mission of the VLD field is to search for valid lie-detection indicators. Both behaviors, lying and telling the truth, involve cognitive processes such as memory, and executive functions, such as working memory, inhibition, and management of information [3]. Therefore, looking for VLD indicators in the cognitive-science framework has been considered most relevant. This search should be guided by two complementary components: theory and applicability. 

A valid indicator should be first and foremost grounded in theory. While this may sound trivial, it is not always followed in deception research [1,4]. The theory establishes the potential mechanism(s) of an indicator, explaining why it distinguishes lies from truths. This understanding is necessary to predict when and how an indicator should work and, maybe even more importantly, when and how it would not be expected to work. An awareness of an indicator’s theoretical reasoning should also be maintained when examining, developing, or applying it.

In addition to the need for theoretical grounding, a valid indicator should also be applicable. Thus, already at the initial developmental stage, one must consider and examine whether it would ultimately be possible to apply it in practice. For example, in real-world settings, practitioners make decisions on single cases, and liars try to hamper detection attempts. Thus, an applicable indicator should allow a decision on a single case and be resistant to countermeasures. Other applicability considerations are the extent to which the use of an indicator requires time and effort, how many prerequisites are required for its administration, and whether it is appropriate and relevant under various conditions. 

“Richness in detail”, one of the most widely investigated verbal cues and part of several VLD tools (e.g., Reality Monitoring [5], Criteria-Based Content Analysis [6]), offers a good illustration of these two components—theory and applicability. According to this indicator, truth-tellers provide richer accounts than lie-tellers in terms of perceptual (e.g., visions, sounds, tastes, smells, physical sensations) and contextual (e.g., times and locations, order of activities, arrangements of objects in space) details. The theoretical explanation underpinning it is derived from Reality Monitoring (RM) theory [7,8], which describes the process used by individuals to monitor their own memory based on its content qualities. According to RM theory, memories of experienced events (i.e., truthful memories) are characterized by perceptual and contextual attributes because the actual event is experienced through the senses and is embedded in context. In contrast, memories of imagined events (i.e., false memories) are characterized by the cognitive operational attributes that help generate them.

A consideration of the theoretical and applied aspects of the richness-in-detail indicator described above gives rise to four issues that warrant attention. First, one should be aware that its theoretical framework—the RM—is a theory of memory rather than deception. Accordingly, it deals with false memories, where the individual unintentionally provides a false account, and not lies, where the individual intentionally provides a false account with the purpose of deceiving [9]. The RM theory does not consider the intention of lie-tellers to deceive and therefore ignores the possibility by lie-tellers of strategically adding false details to increase the level of richness-in-detail artificially. As such, the richness-in-detail indicator is based on theory, but not on an appropriate one for the purpose of lie detection. This has critical implications for its validity as a deception indicator. 

Second, being a memory theory, RM deals with events that were experienced in the past. As such, richness-in-detail, which may have the potential to assess the credibility of past events, should not be applied to assess the credibility of accounts regarding non-past events such as intentions for future activities or descriptions of organizational structures. At the very least, if one applies richness-in-detail to such non-past events, it is important to recognize that this cannot be founded on RM theory. 

Third, richness-in-detail has been found to be subject to individual differences in verbal style and verbal ability [10], such as those associated with different cultures [11] or genders [12]. These individual differences can therefore impede the ability to determine a threshold for this indicator, such as whether a richness-in-detail score would be high enough to conclude that an interviewee was telling the truth or low enough to conclude that an interviewee was lying. The inability to determine an indicator’s threshold due to individual differences makes it difficult to adjudicate individual cases, which is a necessary condition for applicability in practice and thus a serious concern. Fourth and most critically, richness-in-detail has been found to be sensitive to countermeasures. When research participants were provided with insight into the significance of richness-in-detail and were encouraged to include perceptual and contextual details to appear credible, it was no longer able to differentiate between truths and lies based on the richness-in-detail indicator [12]. 

To summarize, the first two points shed light on the theoretical limitations of the indicator, and the last two points certainly undermine its practical use. Therefore, the recent suggestion to favor richness-in-detail over indicators that show a better theoretical relevancy and higher applicability [13] is troubling. This suggestion was based on a comparison of statistical effect sizes obtained for the indicators. It is true that a high effect size is necessary evidence of an indicator’s efficacy. However, it is certainly not sufficient evidence, since effect size alone is not a reliable guide in the search for effective and applicable indicators. In fact, a statistical approach that overlooks theory and practice requirements diverts rather than promotes the process of examining indicators. 

Another issue is the erroneous association of an indicator with a specific theoretical framework that does not actually support it. A prime example is the unverifiable-details indicator, which is sometimes mistakenly attributed to the Verifiability Approach (VA [14]). The VA is a strategy-based framework for the detection of deception. It proposes that lie-tellers are aware that detail-rich accounts are more convincing but know that the provision of many details puts them at risk because investigators can check the truthfulness of the details provided. This poses a dilemma between two contradicting motives—to include many details so that they appear honest and to avoid providing false details to minimize the chances of being caught. A possible strategy of compromise between these two conflicting motives is to provide details that cannot be verified and avoid those that are easy to check. The indicator derived from the VA is the proportion of verifiable details in the provided account, which is expected to be higher among truth-tellers than lie-tellers. The VA cannot support any prediction regarding the unverifiable details [15], and thus it is misleading to use it as a theoretical framework for this indicator. I am not suggesting that it is impossible to examine an indicator of unverifiable details, but rather that when doing so one should support it with valid theoretical grounding. 

## 3. VLD Coding

Coding is an inherent part of any VLD technique. Once accounts are collected, the occurrence frequency of the indicator is measured, mostly by means of human coding. One problem with this practice is the absence of any standardization for VLD coding [1] (#commentary 6), so that different coders are likely to produce different outcomes. This is problematic for research because it precludes a comparison of different studies and consequently impairs the development and examination of VLD techniques. Moreover, a technique in which the decision depends on the coder’s identity is vulnerable to biases and errors in its future use in practice. 

The first step in discussing a potential solution is to define the problem. In the current case, therefore, it is necessary to initially identify the source of the lack of coding standardization and ascertain whether a variation in results is due to the mere human subjectivity of coders or the implementation of different practices. Inter-judge reliability tests measure the extent to which a measurement is subjective. To assess inter-judge reliability, it is mandatory that VLD research include a second independent coder who codes at least some of the accounts. A high inter-judge reliability between coders indicates a sufficient objectivity in judgments, which means that the differences between the coders should not significantly impact their respective results. Research has supported the possibility of achieving a high inter-judge reliability. For example, in one study, fourteen inexperienced coders, trained together, coded the presence of perceptual and contextual details in true and false accounts [16]. Although the training was short (90 min), moderate intraclass correlation coefficients (ICCs), a measure of inter-judge reliability, were obtained for the coding of frequency counts. It has been shown that experienced coders usually show an even higher inter-judge reliability. For example, Nahari [9] reported an ICC = 0.98 for richness-in-detail, which is an almost perfect reliability, and Leal et al. [17] reported an excellent inter-judge reliability of ICC = 0.92 for total details. This is to say that “objectivity” within each study, where the same coding scheme is used, is achievable in VLD coding. 

In contrast, establishing the objectivity of coding in different studies is much more challenging. The literature indicates that researchers employ different coding schemes. This can be illustrated in several examples arising from my extensive 20-year experience in VLD research and development. First, some researchers code words, but others code information units. For example, in the phrase “there was a map on the table”, there are three details (“map” as a perceptual detail; “on” as a contextual detail; and “table” as a perceptual detail) when counting words. However, when counting information units, there are only two details (“there was a map” as a perceptual detail and “on the table” as a contextual detail). Second, researchers vary in their classification of the interviewee’s subjective interpretations. For example, in some studies, “handsome” might be considered a perceptual detail because it is a visual description, but in others it might not because “handsome” is an inferential attribution rather than a sensory attribution. Third, researchers differ in how they refer to the human source of verifiability. For example, when an interviewee mentions the presence of a friend at an event without mentioning the friend’s name, some researchers count this as a verifiability source, because investigators can use a follow-up question to find out the friend’s name and the interviewee is aware of this. In contrast, other researchers will not count this as a verifiability source because the interviewee refrained from providing the friend’s identity in the first place. It is reasonable to assume that differences in coding schemes are due to diverse interpretations of the theoretical logic underpinning the indicators, which in turn results in a variation in the operational definitions of the indicators and, consequently, different indicator scores for the same cases. 

In addition to varying interpretations, coding schemes also differ in the treatment of irrelevant information. For example, facts (e.g., this restaurant serves fresh fish), descriptions of habits (e.g., every morning I eat two eggs), and reasonings (e.g., I smoked because I was nervous) should not be coded. Such pieces of information do not contribute to understanding the investigated events. Moreover, their provision might be used by lie-tellers as a strategy. Coding such irrelevant information artificially inflates the coding scores and can lead to wrong decisions. 

At this point, we can answer the question regarding the source of the lack of coding standardization. It seems that it derives from different practices (i.e., coding schemes) rather than the human subjectivity of coders. Within a given study, inter-judge reliability can confirm the coding accuracy (and thus objectivity) of the specific coding scheme used. However, in light of the different coding schemes used in different studies, the validity of any coding, regardless of its accuracy according to the theoretical framework of the indicator, is questionable. It is impossible to claim that all the different coding schemes produce a valid and correct theoretical interpretation of the same indicator. It is more likely that at least some of the coding schemes are invalid and fail to achieve the measurement for which they were designed. Obviously, invalid coding undermines the findings of the studies and makes it difficult to generalize and compare them. Therefore, I argue that the primary challenge is to ensure the validity, not the reliability, of the coding scheme. 

Now that the problem has been defined, possible solutions can be considered. In their commentary, Verschuere & Meijer [1] (Commentary #6) proposed three means for improving VLD coding. Each can be considered in terms of coding validity, as described earlier. The first suggestion was that researchers specify their coding scheme before data collection and make this information available to others. This is useful mainly for replication purposes, where a further study is designed to confirm the results of the original one. However, it does not resolve the issue of validity. In fact, if the original coding scheme is invalid, its replicated use by other researchers will suffer the same problem. 

The second suggestion was that researchers collaborate to examine the reliability (and validity) of different coding schemes. This is a comprehensive solution that could potentially improve the validity of coding. However, it requires the responsiveness of researchers in the field and their commitment to a long-term process. It is possible that the developers should be the key to defining the coding, each in relation to the technique she or he developed. 

The third suggestion is the automation of coding, which has been indicated as being highly reliable. It is true that computers are more objective and accurate than humans, but it seems that coding automation does not, and perhaps cannot, fulfil the conditions of validity. One reason is that, to date, no such computer program has been developed based on the theoretical VLD approaches. Consequently, the operational definition of the coding does not correspond with the theoretical definitions. Moreover, the method of automated coding (e.g., counting words rather than information units) is not appropriate for VLD coding. Furthermore, computers cannot understand the context of words or consider the background of the accounts (e.g., [1,18]). In private conversations, NLP experts acknowledged that automatic coding that considers context, distinguishes relevant information from facts and habits and identifies implicit repetitions of information was not easy and might be impossible to achieve. Indeed, in a meta-analysis on 79 deception cues from 44 studies where a computer program was used, Hauch et al. [18] concluded that computers are still ineffective lie detectors.

## 4. VLD Research Tracks

VLD research has taken two complementary tracks. The main one addresses the level of development and includes (a) studies that seek indicators, which suggest new approaches for VLD and examine the mechanisms of the indicators; (b) studies that examine the effectiveness of VLD indicators across different contexts (e.g., police interrogation or airport security), target examinees (e.g., suspect, victim, or witness), target populations (e.g., cultures or genders), or interview conditions (e.g., mode of account provision (written or oral), mode of application (real time of the interview or post-interview), or time of application (immediately or with a delay)); and (c) studies that examine the effectiveness of training in applying the indicators. 

Research at the developmental level requires scientific creativity and is the main contributor and catalyst to the advancement of the VLD field. It enhances an understanding of truth-tellers’ and lie-tellers’ verbal behavior and their underlying cognitive mechanisms. At the end of the development and empirical testing, this research also yields VLD techniques and indicators that can be applied in practice. Therefore, the discussion of the search for VLD indicators and VLD coding is primarily relevant to the development-level research track. 

The other track of VLD research focuses on the meta-developmental level and includes direct and conceptual replications as well as meta-analyses and reviews. The role of this research track is to examine and organize the knowledge accumulated by research at the developmental level. This research track is important in examining whether studies’ findings are random or valid, identifying moderating variables, delineating the generalizability of the findings, and defining the general conclusions that can be drawn from the accumulated body of research. 

However, meta-developmental research can be considered a gatekeeper of research in the field. As such, care must be taken to ensure that its processes support, rather than hinder, progress in the field. I would like to raise several issues that warrant attention in this respect. The first issue concerns replications (especially the direct ones). The methodology of VLD studies consists of two main components: (a) collecting true and false accounts under the specific conditions of the specific experiment and (b) coding the accounts according to tested indicators. Therefore, a replication must follow both methodological components. Thus, it is not sufficient to ask for the experiment scenario, instructions for the participants, and measurement tools of the original study, but it is also essential to learn the coding scheme used in the replicated study. A replication study that does not fulfil this requirement misses the mark; consequently, instead of examining and organizing the knowledge in the field, it adds more “noise” to it. 

The second issue is the suggestion to include grey literature (i.e., unpublished studies with limited distribution) in meta-analyses and reviews. The main purpose of including grey literature is to reduce publication bias, which occurs when the publication of a study depends on the significance and direction of effects detected regardless of the quality of the study. Avoiding publication bias is indeed important, but including grey literature bypasses the peer-review process. Peer review, which also serves as gatekeeping, is meant to prevent the publication of low-quality work in scientific journals. It is only reasonable to assume that some of the grey literature was not published because of actual flaws rather than publication bias. Including such low-quality studies in meta-analyses or review papers is liable to distort the insights and conclusions derived from them and consequently lead to an erroneous evaluation of the effectiveness of VLD indicators. Indeed, McAuley et al. [19] observed such a negative influence in the estimation of intervention effectiveness. This suggests that the inclusion of grey literature simply replaces the risk of publication bias with the risk of other biases and errors. The absurdity is that peer review, a necessary step in research at the developmental level, becomes unnecessary precisely at this stage of the meta-developmental research track, which is intended to examine development-level studies. I believe a better solution is to adjust the journal selection and review processes to avoid publication bias in the first place rather than try to correct it in retrospect.

Finally, the discussion of the search for VLD indicators and VLD coding is also relevant to selecting the studies that form the basis of meta-analysis and review studies. Including studies that do not follow the relevant theoretical framework or use invalid coding schemes can lead to incorrect general conclusions, just as the inclusion of grey literature might.

## 5. Conclusions and Future Research Prospects

Based on the meta-research approach, the current paper discussed several issues that challenge both VLD research-developmental and meta-developmental tracks. Addressing some of the issues involves awareness, but dealing with others requires more significant measures. To facilitate these efforts, VLD researchers, as a group with a mutual aim, should try to establish a common language and coding schemes in order to extend the discussion about VLD research qualities and should try to solve its fundamental challenges. Furthermore, sharpening the methodology and reducing the “noise” in the literature could provide solid ground for future research, improving progress and, consequently, leading to better solutions and tools for practitioners.

## Data Availability

Not applicable to this type of paper.
